# Supplementary material for: Compartmentalised mucosal and blood immunity to SARS-CoV-2 is associated with high seroprevalence before the Delta wave in Africa
Source: Commun Med (Lond). 2025 May 16;5:178. doi: 10.1038/s43856-025-00902-x (PMC12084339; doi:10.1038/s43856-025-00902-x)
Supplement: Supplementary file 2 — Supplementary Figs. [file 43856_2025_902_MOESM2_ESM.pdf]

Supplementary Figures

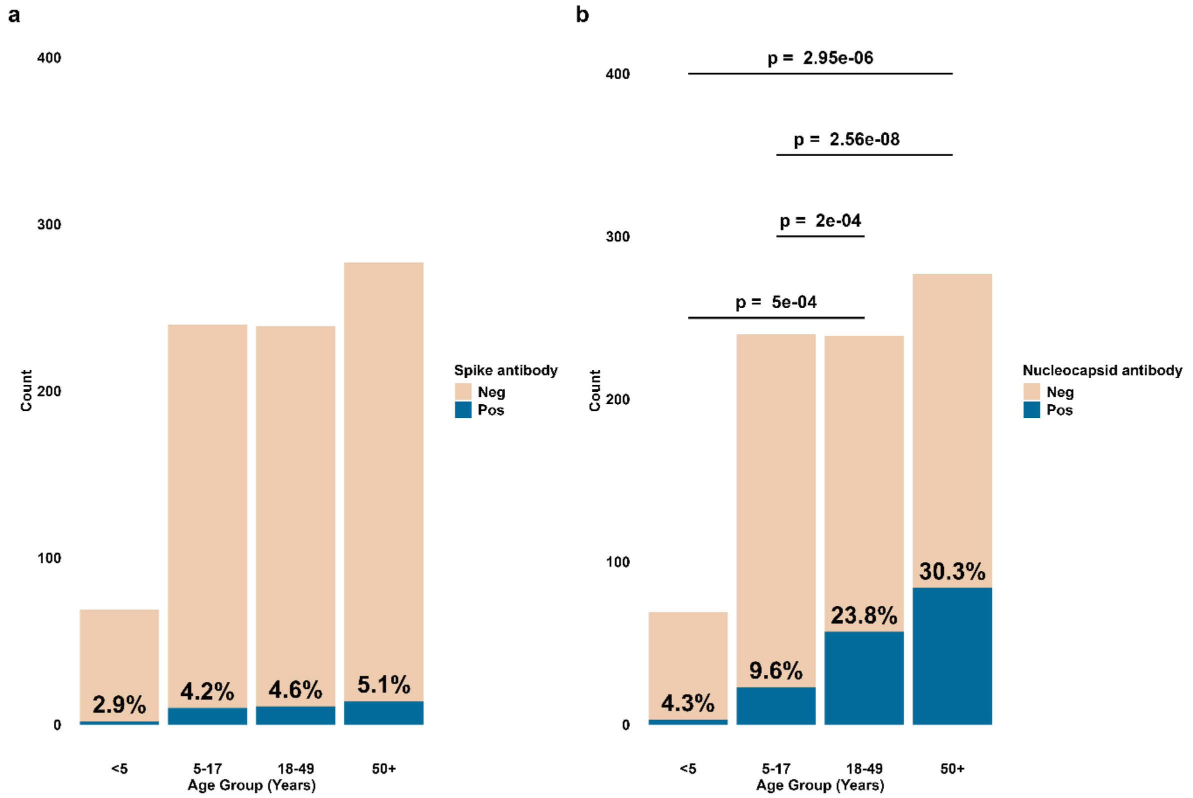

Supplementary Figure 1: Increase in spike and nucleocapsid responses with age in pre-pandemic samples.

The number and percentage of participants that had antibody responses (blue) or no antibody responses (brown) are shown on the histogram for a) Spike and b) Nucleocapsid. Participants were grouped based on age; <5, 5-17, 18-49, and 50+ years. Kruskal-Wallis test was used to assess difference in the number of participants with antibody responses for the different age groups for both proteins in pre-pandemic sera. In cases in which Kruskal–Wallis testing indicated significant differences, post hoc testing using Dunn's test was performed. Correction for multiple comparisons was performed using the Bonferroni–Holm method. The number of samples investigated in each age category was, 69 for <5years, 240 for 5-17 years, 239 for 18-49 years and 277 for ≥50 years. Only significant comparisons are shown on the graph.

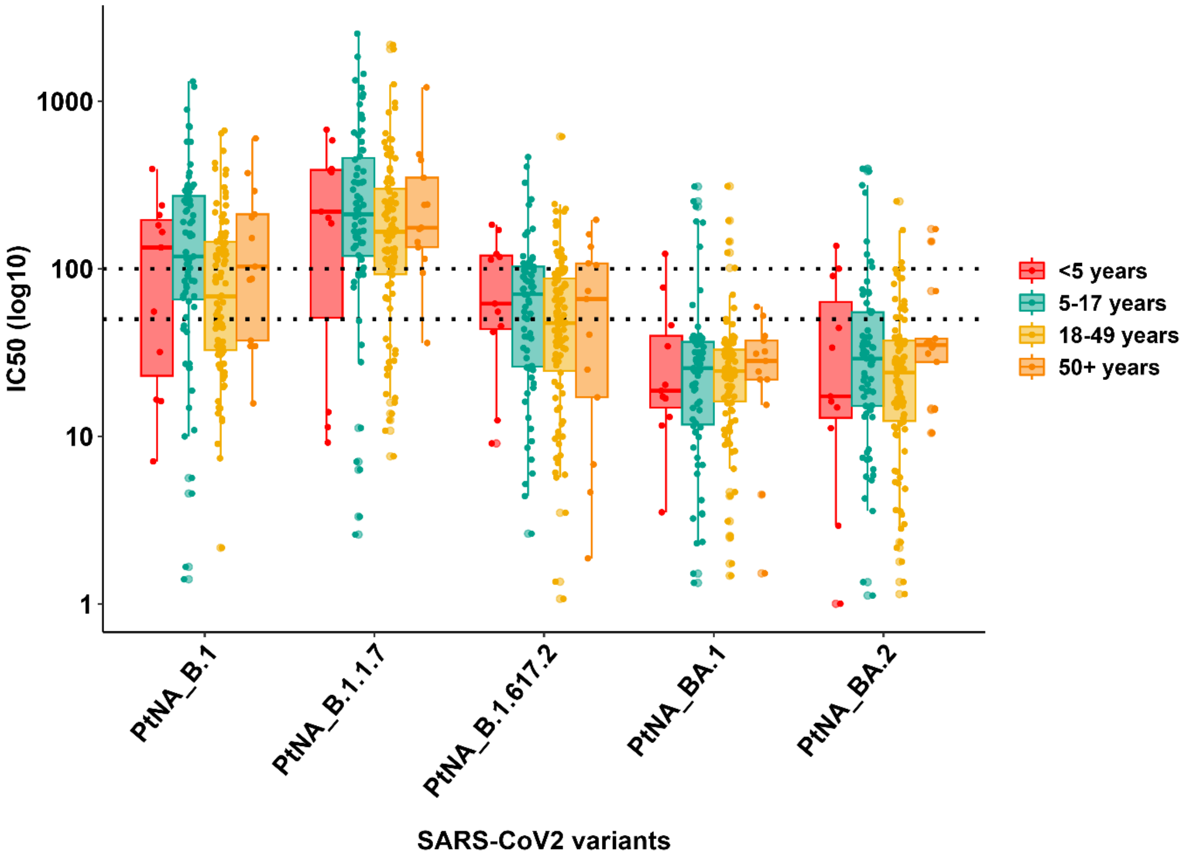

Supplementary Figure 2: Neutralizing antibody responses in participants seropositive to SARS-CoV2.

IC50 (log10) serum titres to ancestral, Alpha (B.1.1.7), Delta (B.1.617.2), Omicron BA.1 and BA.2 variants in SARS CoV-2 seropositive participants <5 (red), 5-17 (green), 18-49 (yellow) and ≥50 years (orange) is shown in the graph. The boxplots denote median IC50, and interquartile range and the dots represent individual responses. The horizontal dotted line at 50 denotes threshold above which neutralizing activity was present for the variants (Ancestral, B.1.1.7, BA.1 and B.A.2) and 100 for the Delta variant (B.1.617.2). Statistical analysis comparing NAb responses to each variant across the different age groups was done using Kruskal-Wallis test. In cases in which Kruskal–Wallis testing indicated significant differences, post hoc testing using Dunn's test was performed. Correction for multiple comparisons was performed using the Bonferroni–Holm method. The lowest serum dilution screened for neutralization was 1:100, with samples not neutralizing at this concentration allocated random values between 1 and 49 for the purpose of statistical analysis and data visualisation. The number of samples investigated was 189 (11 for <5years, 71 for 5-17 years, 94 for 18-49 years and 13 for ≥50 years). Only significant comparisons (p <0.05) are shown on the graph.

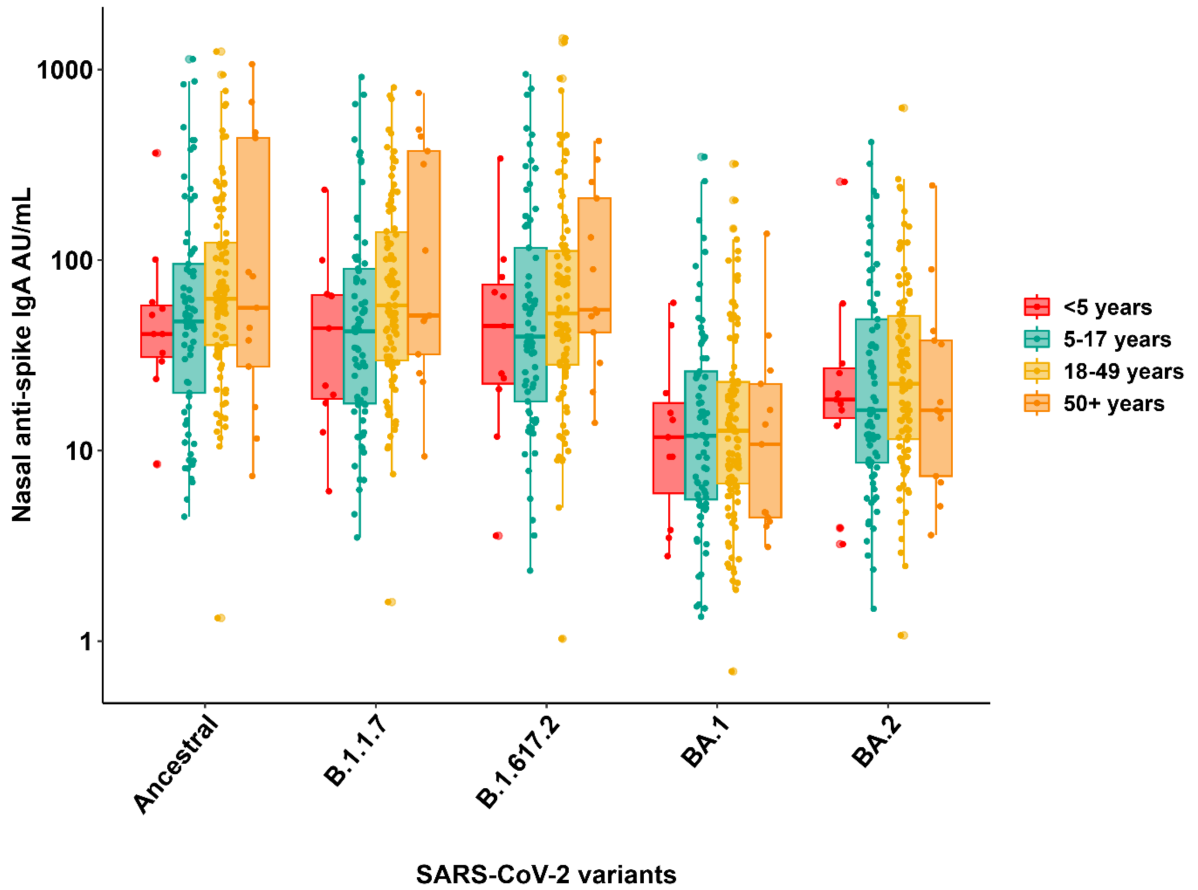

Supplementary Figure 3: Mucosal antibody responses in participants seropositive to SARS-CoV2 spike.

Binding antibody units (AU/mL) to Ancestral, Alpha (B.1.1.7), Delta (B.1.617.2), Omicron BA.1 and BA.2 variants are displayed for SARS CoV-2 spike-seropositive participants <5 (red), 5-17 (green), 18-49 (yellow) and ≥50 years (orange). The boxplots denote median antibody responses and interquartile range, and the dots represent individual responses. Statistical analysis comparing mucosal IgA responses to each variant across the different age groups was done using Kruskal-Wallis test. In cases in which Kruskal–Wallis testing indicated significant differences, post hoc testing using Dunn's test was performed. Correction for multiple comparisons was performed using the Bonferroni–Holm method. The number of samples examined was 196 (11 for <5years, 73 for 5-17 years, 99 for 18-49 years and 13 for ≥50 years).

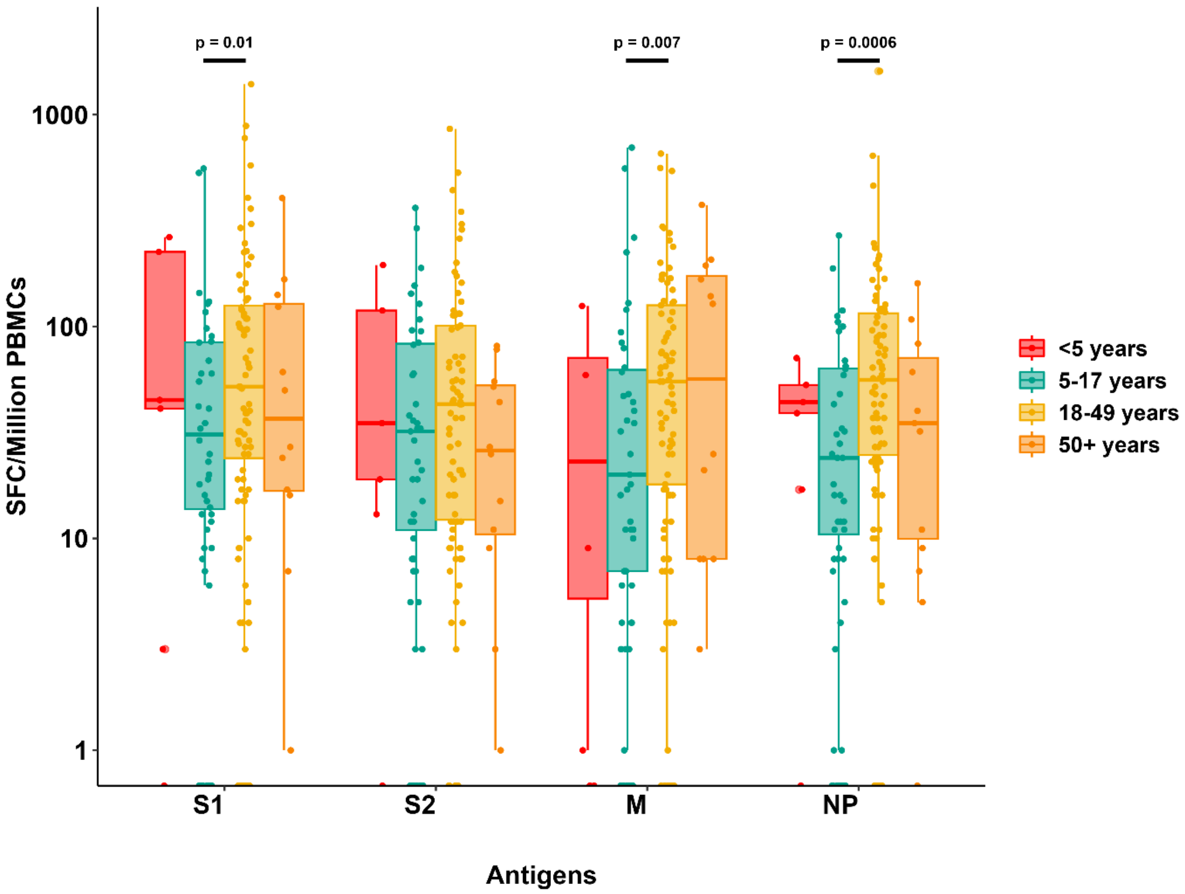

Supplementary Figure 4: T cell responses in participants seropositive to SARS-CoV2.

Interferon-gamma ELISpot responses expressed as spot forming units per million peripheral blood mononuclear cells (PBMC; SFU/mL). (A) T-cell responses to spike S1 and S2 subunits, membrane (M) and nucleocapsid (N) peptide pools is displayed for SARS CoV-2 spike-seropositive participants <5 (red), 5-17 (green), 18-49 (yellow) and ≥50 years (orange). The boxplots denote median and interquartile range and the dots represent individual responses. Statistical analysis comparing T cell responses to each antigen across the different age groups was done using Kruskal-Wallis test. In cases in which Kruskal–Wallis testing indicated significant differences, post hoc testing using Dunn's test was performed. Correction for multiple comparisons was performed using the Bonferroni–Holm method. The number of samples examined was 196 (11 for <5years, 73 for 5-17 years, 99 for 18-49 years and 13 for ≥50 years). Only significant comparisons (p <0.05) are shown on the graph.

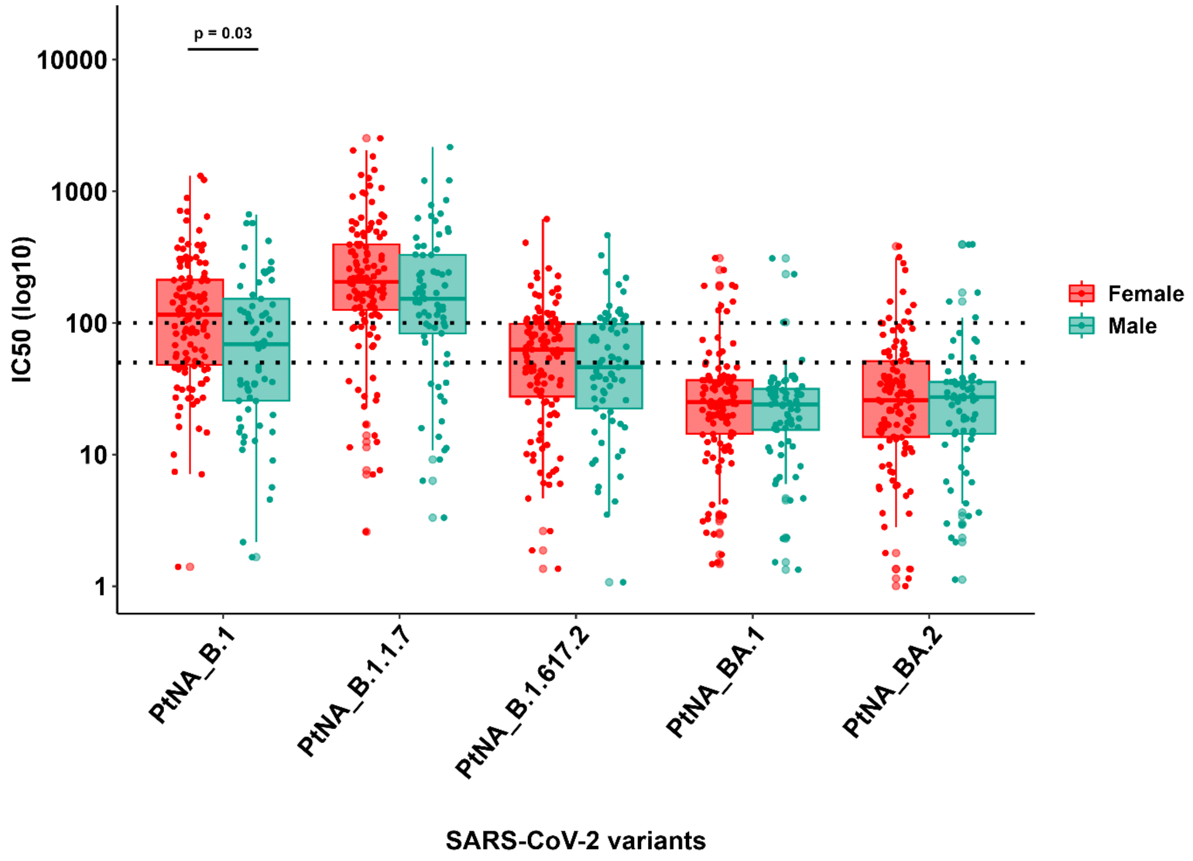

Supplementary Figure 5: Neutralizing antibody responses based on gender in participants seropositive to SARS-CoV2.

IC50 (log10) serum titres to ancestral, Alpha (B.1.1.7), Delta (B.1.617.2), Omicron BA.1 and BA.2 variants in SARS CoV-2 seropositive female (red) and male (green) participants is shown in the graph. The boxplots denote median IC50, and interquartile range and the dots represent individual responses. The horizontal dotted line at 50 denotes threshold above which neutralizing activity was present for the variants (Ancestral, B.1.1.7, BA.1 and B.A.2) and 100 for the Delta variant (B.1.617.2). Statistical analysis comparing NAb responses to each variant across the different age groups was done using Kruskal-Wallis test. In cases in which Kruskal–Wallis testing indicated significant differences, post hoc testing using Dunn's test was performed. Correction for multiple comparisons was performed using the Bonferroni–Holm method. The lowest serum dilution screened for neutralization was 1:100, with samples not neutralizing at this concentration allocated random values between 1 and 49 for the purpose of statistical analysis and data visualisation. The number of females was n=120 and males n=69. Only significant comparisons ($p < 0.05$) are shown on the graph.
