# Supplementary material for: Compartmentalised mucosal and blood immunity to SARS-CoV-2 is associated with high seroprevalence before the Delta wave in Africa
Source: Commun Med (Lond). 2025 May 16;5:178. doi: 10.1038/s43856-025-00902-x (PMC12084339; doi:10.1038/s43856-025-00902-x)
Supplement: Supplementary file 3 — Description of additional supplementary files [file 43856_2025_902_MOESM3_ESM.docx]

Description of Additional Supplementary Files

**File name:** Supplementary data 1

**Description**: Source data for figures
